# ASSOCIATION OF PSYCHOSOCIAL FACTORS WITH MORTALITY IN OLDER FEMALE SURVIVORS OF CANCER

**DOI:** 10.1093/geroni/igac059.2245

**Published:** 2022-12-20

**Authors:** Jennifer King, Kimberly Lind, Kristin Morrill, Cynthia Thomson

**Affiliations:** University of Arizona, Marana, Arizona, United States; University of Arizona, Tucson, Arizona, United States; University of Arizona, Tucson, Arizona, United States; University of Arizona, Tucson, Arizona, United States

## Abstract

Understanding factors associated with survival after a cancer diagnosis among older adults is critical as the population ages and cancer survivorship increases. The purpose of this study was to: (1) identify clusters of postmenopausal cancer survivor characteristics by demographic and lifestyle factors; 2) describe the characteristics of each cluster; and 3) evaluate the association of cluster assignment with survival. Participants from the Women’s Health Initiative (WHI) who reported either a prevalent cancer diagnosis at baseline (n=14294) or were diagnosed with a first primary incident cancer within the first 10 years of WHI (n=12934) were included. Latent class analysis was used to identify survivor clusters using psychosocial variables. Clusters were characterized using descriptive statistics. We tested for differences in cluster characteristics using ANOVA and Chi-square tests as appropriate. Cox proportional hazards regression was used to evaluate the association between cluster and mortality. Prevalent (n=7) and incident (n=9) cancer survivors clusters were identified. Among both (prevalent and incident) sets of clusters, age at WHI baseline, age at menopause, race, ethnicity, income, education, body mass index, diet quality, smoking, alcohol consumption and exercise all differed by cluster (p<.0001 for all). The most racially and ethnically diverse cluster had higher mortality rates compared to the largest most homogenous cluster; hazard ratio (95%CI) 1.30 (1.15, 1.48) and 1.33 (1.16, 1.53), respectively. Understanding how clusters of risk factors influence cancer survival in postmenopausal women will inform future interventions to improve outcomes and reduce health disparities for cancer survivors.

